# Differential Diagnosis of Parotid Tumors on Ultrasound: Interobserver Variability and Examiner-Specific Decision Rules—A Machine Learning Approach

**DOI:** 10.3390/diagnostics16060880

**Published:** 2026-03-16

**Authors:** Lukas Pillong, Ida Ohnesorg, Lukas Alexander Brust, Jan Palm, Julia Schulze-Berge, Victoria Bozzato, Manfred Voges, Adrian Müller, Malvina Garner, Alessandro Bozzato

**Affiliations:** 1 Department of Otorhinolaryngology, Saarland University, Kirrberger Straße 100, 66421 Homburg, Germany; lukas.brust@uks.eu (L.A.B.); victoria.bozzato@uks.eu (V.B.); alessandro.bozzato@uks.eu (A.B.); 2 Medical Faculty, Saarland University, Kirrberger Straße 100, 66421 Homburg, Germany; ida.ohnesorg@uks.eu; 3 Department of Radiation Oncology, Saarland University Medical Center, Kirrberger Straße 100, 66421 Homburg, Germany; jan.palm@uks.eu; 4 Department of Anesthesiology and Intensive Care Medicine, Saarland University Medical Center, Kirrberger Straße 100, 66421 Homburg, Germany; julia.schulze-berge@uks.eu; 5 Independent Researcher, Ober der Trift 39, 66386 St. Ingbert, Germany; manfred.voges@web.de; 6 Department of Computer Science and Microsystems Technology, University of Applied Sciences Kaiserslautern, Amerikastraße 1, 66482 Zweibrücken, Germany; adrian.mueller@hs-kl.de; 7 Department of Neuroradiology, Saarland University Medical Center, Kirrberger Straße 100, 66421 Homburg, Germany; malvina.garner@uks.eu

**Keywords:** ultrasound, parotid gland tumors, machine learning, decision trees, interobserver variability

## Abstract

**Background/Objectives:** Noninvasive differentiation of parotid gland tumors remains challenging despite ultrasound being the primary imaging modality for salivary gland lesions. Given its examiner dependence, improving diagnostic consistency and transparency is crucial. We quantified interobserver variability in parotid ultrasound, modeled examiner-specific decision patterns using machine learning surrogates, and tested whether surrogate complexity relates to examiner performance. **Methods:** In this retrospective, single-center study, six examiners independently rated ultrasound images of 149 parotid tumors using predefined descriptors. Performance was summarized using accuracy and the area under the receiver operating characteristic curve (AUC), with 95% confidence intervals (CIs). AUCs were compared using DeLong tests (Holm-adjusted). Interobserver agreement was assessed using pairwise Cohen’s and global Fleiss’ κ. For each examiner, a decision-tree surrogate was trained from structured descriptors and clinical metadata to reproduce examiner labels and visualize decision pathways; performance was estimated by 5-fold cross-validation. **Results:** Examiner accuracy ranged from 63.5% to 90.5% and AUC from 0.66 to 0.89 (best 0.89, 95% CI 0.83–0.95); the best performer exceeded the two lowest performers (*p* < 0.001). Agreement was higher for objective descriptors (size: κ = 0.57–0.97) than for subjective descriptors (echogenicity: κ = 0.11–0.79). Surrogate decision-tree accuracy versus histopathology ranged from 57.2% to 80.0% for unpruned and from 65.1% to 76.5% for pruned models, with high coverage (95.3–98.7%). Tree complexity showed no consistent association with examiner performance. **Conclusions:** Parotid ultrasound shows substantial interobserver variability. Interpretable surrogates can approximate individual labeling behavior from structured descriptors and clinical metadata, making examiner-dependent decision patterns explicit.

## 1. Introduction

Salivary gland tumors account for approximately 3–6% of head and neck neoplasms (incidence: 0.4–13.5/100,000 annually) [1]. The World Health Organization (WHO) distinguishes over 30 histologic subtypes, most of which are benign (80%) [2]. Accurate preoperative characterization remains difficult due to histologic heterogeneity and overlapping imaging features. As prognosis and treatment differ significantly depending on tumor type, surgical excision remains the diagnostic and therapeutic gold standard. However, parotid surgery carries risks, particularly facial nerve injury, with significant functional and aesthetic consequences [3,4,5]. This underscores the need for reliable, non-invasive imaging tools to differentiate between benign and malignant lesions. Ultrasound is preferred as a first-choice imaging modality due to its real-time capability, availability, lack of radiation, and cost-effectiveness. Multiparametric approaches, including B-mode imaging, color-coded duplex sonography (CDS), contrast-enhanced ultrasound (CEUS), and sonoelastography, have shown promising diagnostic performance [6,7,8,9,10,11]. However, the differential diagnosis of parotid lesions remains challenging, as benign and malignant entities show substantial overlap in conventional B-mode and color Doppler features [12]. This limitation is not restricted to ultrasound. Benign and malignant salivary gland masses can overlap in appearance on MRI [13,14] and CT [15], which can limit reliable characterization using these cross-sectional imaging techniques in routine practice. Human interpretation of imaging is influenced by dual-process cognition: intuitive, experience-based decisions often rely on heuristics, which can introduce cognitive bias [16,17,18]. In examiner-dependent modalities such as ultrasound, these biases may lead to interobserver variability. Accordingly, structured approaches have been proposed to improve the standardization and reproducibility of head and neck ultrasound reporting [19,20,21], and a parotid ultrasound imaging reporting system (PIRADS) has been suggested to support consistent risk stratification, yet variability remains a relevant challenge [22]. A deeper understanding of individual diagnostic strategies could mitigate such variability and inform both training and algorithm design. Recent studies suggest that artificial intelligence (AI) models based on clinical, sonographic, and radiomic features can outperform human assessments [23,24,25]. However, many of these models function as “black boxes,” lacking transparency and explainability. This raises ethical concerns regarding accountability and fairness, particularly when human oversight is limited [26,27,28]. Moreover, comparative AI-versus-clinician studies in medical imaging have been criticized for limitations in design and reporting, as well as a risk of bias, underlining the need for cautious interpretation of performance claims [29].

In this study, we take a complementary approach: rather than replacing human expertise with opaque predictors, we use machine learning surrogates to make examiner-specific diagnostic decision patterns explicit and inspectable. These surrogates were intended as a descriptive interpretability tool. We aimed to (a) quantify interobserver variability across predefined ultrasound descriptors and dichotomous classification, (b) reproduce and visualize individual decision patterns, and (c) determine whether surrogate complexity is associated with examiner-level diagnostic performance.

## 2. Materials and Methods

### 2.1. Study Design

This retrospective, single-center study was approved by the local ethics committee (approval number 83/21) and conducted in accordance with the Declaration of Helsinki. Informed consent was obtained from all participants. Patients with parotid gland tumors treated at our ENT department between 2015 and 2023 were identified from institutional records and screened for eligibility.

### 2.2. Patient Cohort and Ultrasound Image Acquisition

**Figure 1:**
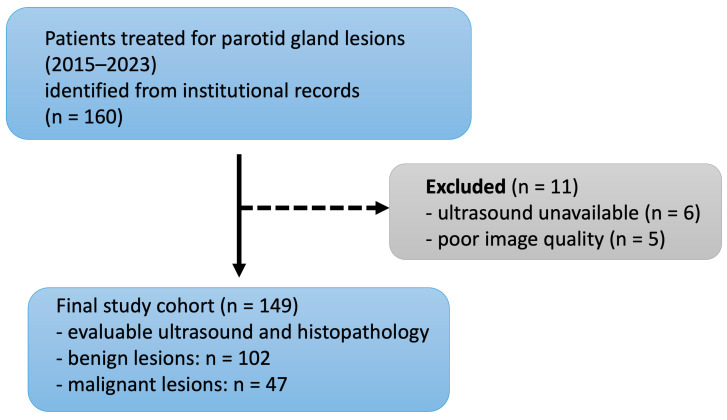
Flowchart of patient selection from initial retrieval to the final study cohort.

Of 160 patients, 11 were excluded due to missing or poor-quality ultrasound. Image quality was assessed by an experienced head and neck radiologist (blinded to clinical and histopathological information) based on predefined criteria, including completeness of the image set, visibility of lesion boundaries, absence of artifacts, and adequate contrast and resolution for morphological assessment (Figure 1).

All ultrasound examinations had been acquired in routine clinical settings using a high-resolution ultrasound system (Acuson S2000, Siemens Healthineers, Erlangen, Germany) equipped with linear transducers (9–14 MHz). Although minor differences in presets and image acquisition protocols existed between operators, all images included in the study were reviewed for diagnostic sufficiency and adherence to general acquisition standards (e.g., lesion-centered orientation, representative longitudinal and transverse planes). No retrospective harmonization of image settings was performed.

### 2.3. Examiners

Six examiners from different specialties and levels of experience independently evaluated the ultrasound images:

Examiner 1: ENT specialist (DEGUM Level III), >20 years’ experience in head and neck ultrasound.
Examiner 2: ENT specialist (DEGUM Level II), 19 years’ experience.
Examiner 3: ENT specialist, 6 years’ experience.
Examiner 4: Radiation oncologist, >20 years’ experience in head and neck imaging.
Examiner 5: Final-year medical student, 1 year of experience in head and neck imaging.
Examiner 6: Neuroradiologist, 7 years’ experience in head and neck imaging.

All examiners received a standardized evaluation guide based on a literature-informed catalog of relevant ultrasound features for parotid gland tumors. The guide was developed prior to the study and structured as tumor-specific profiles derived from a focused literature review [9,13,15,30,31,32,33,34,35,36,37,38,39,40,41,42,43,44,45,46,47,48]. It included morphologic descriptors frequently reported in the diagnostic assessment of benign and malignant salivary gland tumors. A detailed version of the evaluation guide, with literature-backed feature profiles for each tumor entity, is provided in Supplementary File S1. Examiners were aware they were participating in a research study; however, they were not informed of the specific study endpoints. They rated cases independently on retrospective image sets using the standardized evaluation guide and without feedback on their performance or access to other examiners’ assessments.

### 2.4. Evaluation

Evaluations were recorded using structured drop-down spreadsheets:

- Boundary: sharp vs. unclear
- Contour: oval, round, polycyclic
- Echogenicity: anechoic, hypoechoic, hyperechoic, heterogeneous
- Internal texture: homogeneous vs. inhomogeneous
- Vascularization: central, peripheral, diffuse
- Acoustic phenomena: posterior enhancement, shadowing, multiple, none
- Lesion size: <1 cm, 1–2 cm, >2 cm (This categorization was chosen to reflect routine sonographic reporting, reduce rater burden when assessing static image sets, and support interpretable surrogate modeling. Cut-offs were selected after reviewing the size distribution in the dataset and to capture clinically plausible strata for examiner estimation.)
- Number of lesions: solitary vs. multiple

Clinical metadata (age, sex, tumor history, facial nerve function, smoking status, lymph node status) were provided as additional structured input variables because ultrasound interpretation in routine practice is typically contextualized by referral information and clinical examination. All examiners were blinded to the histopathological outcome and independently classified each case as benign or malignant. Representative examples illustrating sonographic variability are shown in Supplementary Figure S1.

The standardized reporting interface was based on the aforementioned evaluation guide. The underlying sonographic descriptors were drawn from prior studies and structured to cover typical sonomorphologic features of both benign and malignant parotid tumors (Supplementary File S1).

### 2.5. Machine Learning Surrogate Modeling (Decision Tree Modeling)

An analysis workflow was implemented in KNIME (version 5.4.1; KNIME AG, Zürich, Switzerland) to model examiner-specific diagnostic decision patterns using decision-tree learning (Supplementary Figure S2). We selected decision trees as examiner-specific surrogates because they yield an inherently interpretable hierarchy of conditional (“if–then”) rules that can be directly inspected and compared across examiners, matching our goal of externalizing examiner-dependent decision patterns rather than maximizing predictive performance [49,50].

For each examiner, the surrogate model was trained to approximate the examiner’s dichotomous label (benign vs. malignant) using predefined ultrasound descriptors and clinical metadata. Histopathology was never used as a predictor to prevent information leakage. Because the training target is the examiner label, we evaluated two complementary endpoints:

(a) Surrogate fidelity, defined as agreement between surrogate predictions and the corresponding examiner labels (i.e., how well the surrogate reproduces the examiner’s labeling behavior).
(b) Clinical performance vs. histopathology, defined as surrogate predictions compared with histopathology, which contextualizes examiner-derived labeling behavior relative to the reference standard.

We used stratified k-fold cross-validation (k = 5) stratified by the examiner’s binary label (class column). For each fold, the decision tree was trained on k − 1 folds and evaluated on the held-out fold. Out-of-fold predictions were aggregated across folds to compute fidelity and clinical performance metrics. Two tree variants were evaluated per examiner: an unpruned tree and a pruned tree (MDL-pruning as implemented in the KNIME Decision Tree Learner). Missing predictions (if present) were excluded from metric computation, and coverage (proportion of cases with non-missing predictions) was reported.

We trained a tree per examiner on the complete dataset using the same preprocessing and hyperparameters as in the cross-validation setting. These final trees were used for visualization and qualitative inspection of decision pathways, whereas reported performance metrics are based on out-of-fold cross-validated predictions.

### 2.6. Statistical Analysis

All statistical analyses were performed using R (version 4.4.0; R Foundation for Statistical Computing, Vienna, Austria) with the package Version (“pROC”) ‘1.19.0.1’ and package Version (“binom”) ‘1.1.1.1’. A two-sided *p*-value < 0.05 was considered statistically significant.

For the examiner’s diagnostic performance, sensitivity, specificity, accuracy, positive predictive value (PPV), and negative predictive value (NPV) were calculated. Receiver operating characteristic (ROC) curves and corresponding area under the curve (AUC) values with 95% confidence intervals (CI) were calculated for each examiner. Confidence intervals were derived using DeLong’s method [51]. Pairwise comparisons of AUCs were conducted using DeLong’s test. Comparisons of sensitivity and specificity between examiner pairs were performed using McNemar’s test [52] with Holm adjustment for multiple comparisons.

Interobserver agreement for each sonographic descriptor and dichotomous classification was quantified using pairwise Cohen’s kappa (κ) across all examiner pairs [53], using pairwise complete cases for each examiner pair and descriptor. To summarize agreement across all six examiners, we additionally calculated Fleiss’ κ for each descriptor using complete cases across all raters. Ninety-five percent confidence intervals for Fleiss’ κ were derived by nonparametric bootstrap resampling. Kappa statistics were reported as effect-size measures of agreement (with 95% confidence intervals where applicable); because κ estimates were used descriptively (i.e., without *p*-value-based hypothesis testing), no multiple-comparison correction was applied to agreement analyses. For descriptive interpretation, κ values were categorized according to Landis and Koch as a heuristic guide [54]; given the dependence of κ on prevalence and marginal distributions, thresholds were interpreted contextually.

To assess robustness and potential overfitting of surrogate trees, we compared pruned versus unpruned decision-tree variants using paired out-of-fold predictions for each examiner. Analyses were performed for both (a) surrogate performance versus histopathology and (b) surrogate fidelity versus examiner labels. Differences in classification errors were tested using exact McNemar tests with Holm correction across examiners to control the family-wise error rate; coverage was reported to account for missing predictions. In addition to accuracy and threshold-based metrics, Cohen’s κ was reported as a chance-corrected agreement measure for both surrogate endpoints. For unpruned trees, cases with missing surrogate predictions were excluded from κ estimation. Ninety-five percent confidence intervals for surrogate κ were obtained by nonparametric bootstrap resampling of cases.

## 3. Results

### 3.1. Study Population

As shown in Figure 1, 149 of 160 identified patients were included in the study; 102 (68.5%) had a benign and 47 (31.5%) had a malignant salivary gland lesion. Baseline characteristics are summarized in Table 1.

### 3.2. Diagnostic Performance

**Figure 2:**
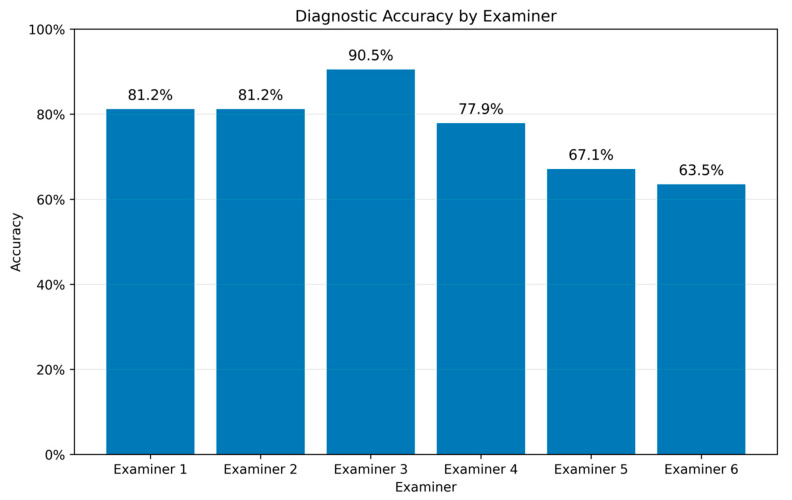
Accuracy of the diagnostic performance for each examiner (Examiner 1: 81.2%; Examiner 2: 81.2%, Examiner 3: 90.5%; Examiner 4: 77.9%; Examiner 5: 67.1%; Examiner 6: 63.5%). Bars indicate the proportion of correct assessments relative to the definitive histopathological diagnosis.

Diagnostic accuracy differed across examiners, ranging from 63.5% (Examiner 6) to 90.5% (Examiner 3) (Figure 2).

**Figure 3:**
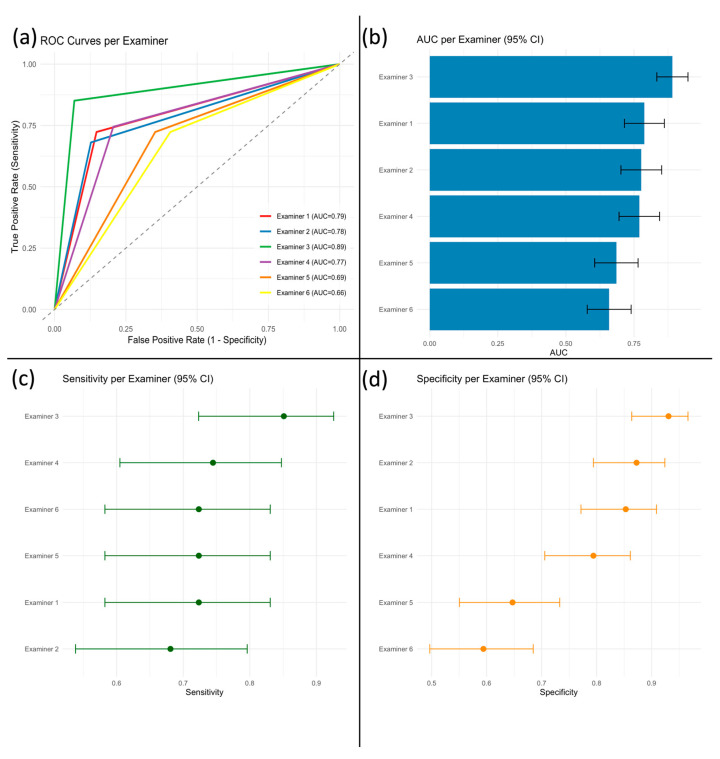
Diagnostic performance of six examiners in classifying parotid tumors by ultrasound. (**a**) Receiver operating characteristic (ROC) curves for each examiner; the diagonal dashed line indicates chance level (AUC = 0.5). (**b**) Area under the ROC curve (AUC) with 95% confidence intervals. (**c**) Sensitivity (true positive rate) and (**d**) specificity (true negative rate) for each examiner, both with 95% confidence intervals.

Figure 2 shows the proportion of correct binary classifications (benign vs. malignant) relative to the definitive histopathological diagnosis. A complete summary of diagnostic performance metrics is provided in Supplementary Table S1. Examiner 3 demonstrated the highest overall performance (accuracy 90.5%) and the highest AUC (0.89; 95% CI: 0.83–0.95) (Figure 3a,b). The remaining examiners showed more modest differences, with substantial overlap of confidence intervals (AUCs 0.66–0.79), indicating limited evidence for meaningful separation based on point estimates alone. Between-examiner differences were further evaluated using paired comparisons, which confirmed that meaningful separation was primarily driven by Examiner 3. Holm-adjusted DeLong tests confirmed that Examiner 3 achieved significantly higher AUC than Examiners 5 and 6 (both *p* < 0.001), whereas other pairwise AUC comparisons were not statistically significant.

Sensitivity estimates overlapped across all examiners (Figure 3c), and Holm-adjusted McNemar tests showed no significant differences in sensitivity between examiner pairs. In contrast, specificity was reduced in Examiners 5 and 6 (Figure 3d), and Holm-adjusted McNemar tests confirmed significantly lower specificity compared with Examiners 1–4 (all *p* < 0.02).

An overview of diagnostic metrics is provided in Figure 3.

In addition to the confusion-matrix metrics reported above, a descriptive summary of misclassification patterns stratified by histopathological entity is provided in Supplementary Table S2.

### 3.3. Interobserver Agreement

Interobserver agreement varied across sonographic features and examiners. Highest agreement was observed for tumor size (κ = 0.57–0.97) and malignancy assessment (benign vs. malignant) (κ = 0.31–0.94), with Examiners 1 and 2 demonstrating near-perfect agreement (κ = 0.97 for size, κ = 0.94 for malignancy assessment (benign vs. malignant)).

Moderate to substantial agreement was found for boundary (κ = 0.43–0.83), contour (κ = 0.29–0.76), vascularization (κ = 0.21–0.87), and number of tumors (κ = 0.30–0.87). Qualitative features showed greater variability, with echogenicity (κ = 0.11–0.79), acoustic phenomena (κ = 0.28–0.87), and texture (κ = 0.33–0.79) reaching only slight to moderate agreement in some examiner pairs.

Global multi-rater agreement across all six examiners (Fleiss’ κ) was highest for size (κ = 0.77, 95% CI 0.71–0.82) and boundary (κ = 0.62, 95% CI 0.55–0.69), moderate for malignancy assessment (benign vs. malignant) (κ = 0.48, 95% CI 0.40–0.56) and texture (κ = 0.49, 95% CI 0.41–0.57), and lowest for echogenicity (κ = 0.27, 95% CI 0.20–0.33) (Supplementary Table S3).

Overall, quantitative parameters, such as lesion size, showed high reproducibility, whereas more subjective, qualitative features (e.g., texture, echogenicity) were prone to interobserver variability.

**Figure 4:**
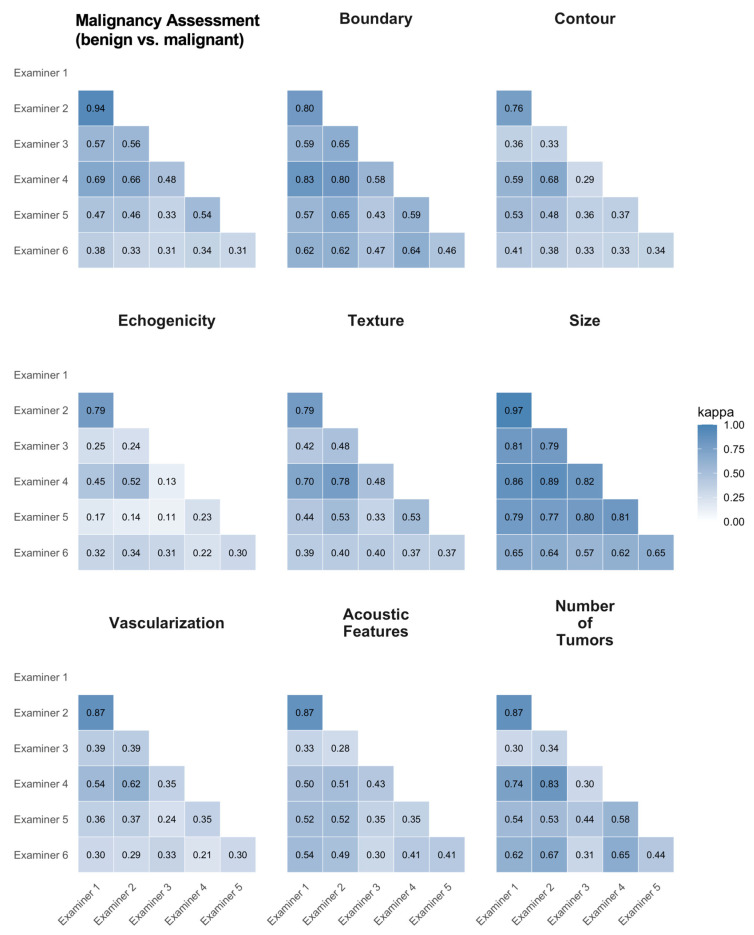
Pairwise inter-observer agreement for nine sonographic features, expressed as Cohen’s κ. κ was calculated as unweighted Cohen’s κ using pairwise complete cases (*n* = 147–149, depending on the examiner pair). Each panel displays a lower-triangular heatmap of κ values for all examiner pairs (Examiner 1–6), with cells color-coded from light blue (low agreement) to dark blue (high agreement) and κ values shown numerically. From left to right and top to bottom, panels represent: Malignancy Assessment (benign vs. malignant), Boundary, Contour, Echogenicity, Texture, Size, Vascularization, Acoustic Features, and Number of Tumors.

A detailed breakdown of interrater statistics is shown in Figure 4.

### 3.4. Examiner-Specific Surrogate Models (Decision Tree Analysis)

For each examiner, an examiner-specific decision tree surrogate model was trained using the structured ultrasound descriptors and clinical metadata to approximate the examiner’s binary labeling behavior (benign vs. malignant). The resulting surrogate trees made examiner-specific decision pathways explicit and enabled systematic comparison of feature prioritization, particularly within the upper tree structure.

#### 3.4.1. Tumor History-Rooted Trees (Examiners 1–2)

Examiners 1 and 2 produced highly similar surrogate trees, with Tumor History as the root split and identical top-level branching behavior (Supplementary Tables S4 and S5; Supplementary Figures S3 and S4). In both models, the absence of tumor history (Tumor History = no) constituted the dominant branch (114/149 cases, 76.5%), followed by Boundary as the next split. Conversely, the presence of tumor history (Tumor History = yes) comprised a smaller branch (35/149 cases, 23.5%) and was followed by Vascularization. Downstream refinements differed across branches and included additional sonographic descriptors and clinical metadata such as nicotine consumption and acoustic features (Examiner 1) and facial nerve palsy within the unclear Boundary-branch (Examiner 2).

#### 3.4.2. Boundary-Rooted Surrogate Trees (Examiners 3–6): Shared Early Heuristics and Branch-Specific Divergence

Four examiners (Examiners 3–6) employed Boundary as the root split, indicating a shared early heuristic centered on lesion margins (Supplementary Table S4, Supplementary Figures S5–S8). Despite this common starting point, the subsequent branching patterns differed in a branch-specific manner. For the sharp-Boundary-branch, Examiners 3–5 most frequently selected Tumor History as the next split, whereas Examiner 6 diverged by selecting Facial Nerve Palsy (Supplementary Table S4). For the unclear Boundary-branch, follow-up splits were less consistent across examiners, with Examiner 3 splitting on Contour, Examiners 4–5 on Facial Nerve Palsy, and Examiner 6 on Acoustic Features (Supplementary Table S4).

Pairwise similarity analyses of the upper tree structure (depth 0–2) revealed two stable examiner clusters (Examiner 1/2 and Examiner 4/5). In contrast, Examiner 6 shared only limited upper-branch similarity with the other boundary-rooted trees, consistent with early divergence in follow-up splits (Supplementary Table S5). Path-level agreement was highest in the upper structure (depth 0–1) and decreased substantially at depth 2, indicating shared initial heuristics but increasing examiner-specific refinement in downstream branches (Supplementary Table S5).

#### 3.4.3. Tree Complexity, Pruning, and Surrogate Model Performance

Structural complexity metrics extracted from the Decision Trees demonstrated substantial variability across surrogate trees (Supplementary Table S6). Examiner 6 exhibited the largest tree by node/leaf counts, whereas several other examiners showed comparable maximum depth ranges. Formal correlation analyses between surrogate tree complexity and examiner diagnostic performance relative to histopathology did not demonstrate a consistent association between global tree size (node/leaf counts) and performance; depth-based measures showed at most an exploratory trend, most pronounced for specificity (Supplementary Table S7). Pruning increased model coverage to 100% for all examiners, whereas unpruned trees produced missing predictions in a small subset of cases (coverage 95.3–98.7%). After Holm correction, a significant improvement of pruned versus unpruned performance against histopathology was observed only for Examiner 6; for surrogate fidelity against examiner labels, no significant differences in paired classification errors were detected across examiners (Supplementary Table S8).

## 4. Discussion

### 4.1. Surrogate Modeling Externalizes Examiner-Specific Decision Patterns

In this retrospective, single-center study of 149 parotid tumors, we observed substantial interobserver variability in descriptor assessments and a wide range of diagnostic performance across examiners. To make examiner-dependent labeling behavior transparent and comparable, we trained examiner-specific interpretable surrogate models (decision trees) based on structured ultrasound descriptors and clinical metadata. These surrogates provide data-driven approximations of each examiner’s binary classification behavior (benign vs. malignant) and allow direct inspection of feature prioritization and branch-specific decision pathways. Importantly, the surrogate trees should not be interpreted as direct representations of human cognitive processes; rather, they summarize how the available structured inputs map to an examiner’s observed labels in this cohort.

This approach is clinically relevant because ultrasound is widely used as a first-line imaging modality for salivary gland lesions [40,55], and its interpretation is known to be operator-dependent, with training and experience influencing diagnostic consistency [56,57]. By externalizing examiner-specific rules into inspectable tree structures, the method enables comparison of shared early heuristics and downstream divergence, and may support structured teaching, reporting standardization, and targeted feedback.

#### 4.1.1. Differential Predictive Value and Prioritization of Features in the Surrogate Trees

Analysis of examiner-specific surrogate trees revealed shared upper-level decision heuristics and branch-specific divergence. Across examiners, the root split most frequently relied on Boundary (4/6 examiners), whereas Tumor History served as the alternative root discriminator (2/6 examiners). This suggests that, within the structured descriptor set and the present cohort, lesion margin characteristics and clinical history information form common initial “triage” features in examiner labeling behavior.

Beyond the root level, conditional agreement was most apparent in the first branching layer. For example, within the Boundary-branch, several examiners selected Tumor History as the next discriminator. In contrast, the unclear Boundary branch was followed by different features across examiners (e.g., Contour, Facial Nerve Palsy, or Acoustic Features). Agreement decreased markedly at deeper levels, indicating increasing examiner-specific refinement rather than a single shared downstream hierarchy. These findings are consistent with the notion that ultrasound interpretation is operator-dependent and shaped by training and local conventions [56,57]

Importantly, some descriptors appeared as early discriminators only in specific examiners (e.g., Contour in one boundary-rooted tree; Facial Nerve Palsy in others), highlighting that “feature importance” is conditional on the path context and may not generalize across raters. Echogenicity and vascularization tended to appear as downstream refinements in several trees, while clinical metadata (tumor history, facial nerve palsy, nicotine consumption) were sometimes used early in specific branches.

In line with prior work applying interpretable models and quantitative approaches for parotid tumor characterization [23,25,55], our approach does not aim to optimize a single best-performing classifier but to externalize and compare examiner-dependent decision patterns, which may support targeted teaching, descriptor calibration, and structured reporting initiatives [19,20,21,58,59].

#### 4.1.2. Surrogate Tree Structure, Complexity, and Examiner Performance

A key goal of the present study was to formally assess whether surrogate tree complexity relates to examiner diagnostic performance. Using PMML-derived complexity metrics (e.g., total number of nodes, number of leaves, maximum depth, and record-count-weighted mean leaf depth), we statistically tested associations with examiner performance measures relative to histopathology. These analyses did not demonstrate a consistent relationship between global tree size (node/leaf counts) and examiner performance. Depth-based measures showed, at most, an exploratory negative trend, most pronounced for specificity. Given the small number of examiners, these findings should be interpreted as hypothesis-generating rather than confirmatory.

In parallel, the structural agreement analyses provide a complementary perspective that is not captured by scalar complexity metrics alone. The upper-tree topology suggests shared initial heuristics (Boundary vs. Tumor History as root splits), whereas deeper branching reflects increasing examiner-specific refinement. This pattern supports a cautious interpretation: differences between examiners may be driven less by entirely different starting rules and more by branch-specific downstream decisions, which can influence operating points (e.g., low specificity/high false-positive rates) in clinically relevant ways.

Pruning analyses further indicated that model structure interacts with practical usability. Pruning improved coverage (eliminating missing predictions) across all examiners and yielded a statistically detectable improvement in performance against histopathology only for one examiner, while surrogate fidelity against examiner labels did not change significantly. This reinforces that surrogate trees should be interpreted primarily as descriptive tools that approximate labeling behavior, not as causal models of human reasoning. The apparent complexity of a surrogate tree may reflect multiple factors, including label variability, feature correlations, and limited sample size, rather than “better” or “worse” diagnostic reasoning [49,60,61]. While we focused on decision trees to externalize examiner-specific pathways, other inherently interpretable model classes could be considered, including sparse logistic regression, generalized additive models (GAMs), and rule-list/rule-set approaches. These methods provide complementary forms of interpretability (e.g., global coefficients or smooth feature effects), but they do not always yield a single, easily comparable hierarchical pathway across examiners. In the present study, decision trees offered a pragmatic representation that supports both visual inspection and path-level agreement analyses of early heuristics and downstream divergence. Future work could compare surrogate fidelity, stability, and complexity–performance relationships across different interpretable model classes under identical feature sets and data splits [49,50,60].

Finally, the early use of clinical context variables (e.g., tumor history) in some trees may reflect efficient heuristics but also raises the possibility of context-driven decision tendencies. Such heuristics can support efficient decision-making under time constraints yet may increase susceptibility to systematic bias (e.g., priming effects or premature closure) [16,17,62,63]. In the clinical context of parotid tumors, low-specificity operating points translate into higher false-positive rates, potentially triggering unnecessary downstream work-up and avoidable surgery, whereas high-specificity/low-sensitivity strategies reduce overtreatment at the risk of missed malignancy. Making these examiner-specific trade-offs explicit remains a key advantage of the surrogate modeling approach.

### 4.2. Influence of Experience, Training Environment, and Standardized Training on Agreement Patterns

Interobserver agreement and diagnostic performance varied across examiners. However, given the small number of examiners (*n* = 6) and the monocentric setting, examiner experience level, specialty background, and exposure to structured ultrasound curricula are closely intertwined and cannot be disentangled causally. Therefore, any apparent group differences should be interpreted as descriptive and hypothesis-generating rather than evidence of specialty- or experience-specific effects.

Beyond summary agreement metrics, the surrogate-tree comparison provides an additional perspective on examiner similarity. Pairwise similarity analyses of the upper tree structure revealed two stable examiner pairs with highly similar top-level decision pathways (Examiner 1/2 and Examiner 4/5), consistent with the idea that shared training environments and local conventions may shape both descriptor use and decision rules. More broadly, the trees showed shared early heuristics (e.g., Boundary vs. Tumor History as root discriminators) but increasing branch-specific divergence at deeper levels, suggesting that common initial triage criteria may coexist with heterogeneous downstream refinement strategies.

Although the present study is not designed to test training effects, formally standardized training and structured criteria have been proposed to improve consistency in sonography and reduce interobserver variability [22,57,64,65]. In addition, institutional practice patterns (acquisition presets, reporting conventions, and teaching culture) may have influenced both agreement estimates and the inferred surrogate pathways. Accordingly, the examiner-specific surrogate rules should be interpreted as context-dependent representations of diagnostic behavior within this institutional setting rather than universally transferable decision algorithms.

### 4.3. Clinical Implications: Structured Reporting, Examiner Training, and Explainable AI

Our findings have practical implications for standardization and education in examiner-dependent ultrasound. Structured reporting initiatives in radiology and head-and-neck ultrasound aim to improve completeness, clarity, and comparability of reports and enable downstream reuse of structured data for quality assurance and model development [19,20,21,58,59]. In this context, examiner-specific surrogate trees could complement conventional agreement metrics by revealing which descriptors are prioritized early and where examiner decision pathways diverge.

The PMML-based tree comparison indicates that examiners share upper-level heuristics (e.g., Boundary or Tumor History as root discriminators) while showing increasing divergence in deeper, branch-specific refinements. This suggests two actionable opportunities: (i) emphasizing consistent documentation and calibration of a small set of “high-yield” early descriptors (e.g., margin characteristics and clinical history items), and (ii) identifying descriptors and branch contexts where definitions or use are less aligned across examiners (e.g., follow-up splits after Boundary = unclear). Rather than assuming that one universal feature hierarchy applies to all readers, the surrogate-tree framework explicitly captures conditional feature use (path-dependent prioritization), which may help refine teaching cases and structured templates.

Examiner-specific surrogates may also serve as a tool for targeted training and feedback. Prior work has highlighted the potential of probabilistic and interpretable models in educational settings by making decision structures explicit and enabling case-based review [66]. In our setting, surrogate rules could support feedback discussions such as “which descriptor was decisive for this examiner’s label” and “how does this differ from high-specificity vs. high-sensitivity operating points”. This is particularly relevant because clinically meaningful trade-offs can emerge even when overall accuracy differences are modest; for example, reduced specificity (higher false-positive rates) can lead to unnecessary downstream work-up and potentially avoidable surgery.

Finally, with increasing interest in explainable AI, there is a growing emphasis on designing inherently interpretable models and auditing diagnostic pathways rather than solely reporting final predictions [60]. Examiner-specific surrogates provide a human-interpretable reference that can help (a) identify stable, clinically plausible early heuristics, (b) detect potential shortcut strategies (e.g., early reliance on clinical context), and (c) define which parts of the diagnostic pathway should remain transparent in clinical workflows. Importantly, our approach is intended as a descriptive framework for transparency and hypothesis generation rather than as a deployable diagnostic system.

### 4.4. Limitations

This study has several limitations. First, the monocentric design and limited sample size constrain generalizability. All examinations were acquired within a single institutional workflow and predominantly on one ultrasound platform and protocol, which may encode vendor- and site-specific characteristics and influence descriptor ratings. Examiner backgrounds differed in ultrasound proficiency and training exposure; with only six examiners, specialty, experience, and institutional training are tightly intertwined and cannot be separated causally. Accordingly, subgroup interpretations should be regarded as descriptive.

Second, the surrogate trees were trained to approximate examiner labeling behavior from structured descriptors and clinical metadata. As such, they represent cohort- and context-dependent approximations rather than direct measurements of cognitive reasoning processes. While descriptor collection was standardized, examiner-specific cues not captured by the structured feature set may have been missed. Moreover, the availability, completeness, and reliability of the incorporated clinical metadata may vary across referral settings (e.g., facial nerve palsy is typically clinically well-defined, whereas smoking status and detailed tumor history may be incompletely documented). Surrogate structure can reflect label variability, feature correlations, and limited sample size rather than “thought complexity.” Decision trees are also sensitive to small perturbations and may yield multiple structurally distinct solutions with similar performance; therefore, the displayed trees should be interpreted as a single plausible representation of the mapping from structured inputs to labels in this dataset. Although our primary endpoint was dichotomous classification, the cohort’s case-mix may still have shaped examiner heuristics, and thus the resulting surrogate hierarchies, by emphasizing patterns typical of dominant entities. Accordingly, the extracted decision rules should be interpreted as cohort-dependent and warrant validation in external cohorts with broader and more balanced subtype representation.

Third, diagnostic performance analyses were based on binary examiner decisions without continuous confidence scores. As a result, ROC/AUC estimates reflect discrimination derived from hard classifications rather than full score distributions, and AUC is numerically equivalent to balanced accuracy in this setting. While we used paired statistical tests to compare examiners, these results should be interpreted with this constraint in mind. In addition, agreement metrics such as κ are sensitive to prevalence and marginal distributions and should be interpreted in the context of the observed descriptor and class distributions.

Fourth, pruning and missing predictions highlight a practical modeling limitation: unpruned trees produced missing predictions in a small subset of cases, reducing coverage, whereas pruning achieved complete coverage across examiners. Pruning improved performance against histopathology only for one examiner and did not significantly alter surrogate fidelity against examiner labels, indicating that pruning primarily affected usability and coverage rather than systematically improving decision fidelity.

Finally, no independent external validation cohort was available to assess the stability of examiner-specific surrogate pathways across centers, ultrasound platforms, acquisition protocols, or case mix. Future work should evaluate the framework in multicenter, multi-vendor cohorts and assess the stability of surrogate decision pathways over time and under varying technical conditions. Prospective evaluation in training and quality-assurance settings could test whether case-based feedback using examiner-specific surrogates improves descriptor calibration, interobserver agreement, and clinically relevant operating points. These surrogates should therefore be interpreted as descriptive transparency tools for education and hypothesis generation rather than as deployable diagnostic decision-support systems.

## 5. Conclusions

Accurate ultrasound-based classification of parotid tumors remains challenging, with substantial interobserver variability. Examiner-specific surrogate decision trees based on structured descriptors and clinical metadata made individual decision pathways transparent, revealing shared early heuristics but divergent downstream refinements. This framework can support targeted training, descriptor calibration, and quality assurance, and provides a clinically interpretable reference for explainable AI in parotid ultrasound diagnostics.

## Figures and Tables

**Table 1:** Baseline characteristics with histological subtypes of the parotid gland tumors included in the study cohort.

Histology	*n*	% of Cohort	Age (Mean ± SD, Yrs)	Male *n* (%)	Female *n* (%)
malignant	47	31.5%	69.4 ± 16.4	29 (61.7%)	18 (38.3%)
Warthin tumor	41	27.5%	63.5 ± 10.2	25 (61.0%)	16 (39.0%)
pleomorphic adenoma	34	22.8%	55.1 ± 12.8	15 (44.1%)	19 (55.9%)
sialadenitis	8	5.4%	48.1 ± 21.1	3 (37.5%)	5 (62.5%)
lymph node	7	4.7%	59.4 ± 10.6	2 (28.6%)	5 (71.4%)
cyst	3	2.0%	54.7 ± 24.8	2 (66.7%)	1 (33.3%)
angioma	1	0.7%	30.0 ± 0.0	0 (0.0%)	1 (100.0%)
other entities	8	5.4%	68.5 ± 11.1	5 (62.5%)	3 (37.5%)

## Data Availability

The data are not publicly available due to privacy and ethical restrictions, but de-identified data may be made available from the corresponding author upon reasonable request and subject to approval by the local ethics committee/data protection regulations.

## Supplementary Materials

The following supporting information can be downloaded at: https://www.mdpi.com/article/10.3390/diagnostics16060880/s1, File S1: Literature-Based Ultrasound Profiles of Parotid Tumor Entities. This file summarizes typical ultrasound features (e.g., echogenicity, internal architecture, vascularity) of common and rare parotid tumor subtypes, as reported in the literature. It served as the foundation for the standardized evaluation guide used by all examiners; Figure S1: Representative ultrasound images of parotid gland tumors assessed in this study, illustrating the wide morphological variability encountered in ultrasound imaging of parotid lesions; Figure S2: Schematic overview of the KNIME-based workflow used to model examiner-specific diagnostic strategies via decision tree learning; Figure S3: This examiner-specific surrogate decision tree reproduces Examiner 1’s binary labeling behavior. The root split is Tumor History, followed by branch-specific refinement using Boundary (e.g., sharp vs. unclear) and Vascularization. Downstream splits include additional sonographic descriptors and clinical metadata such as Echogenicity, Acoustic Features, Nicotine consumption, Texture, Contour, and Number of Tumors, yielding an explicit hierarchical pathway for the examiner’s benign vs. malignant calls; Figure S4: Decision-tree surrogate derived from Examiner 2’s assessments. This surrogate decision tree was trained to approximate the binary labels of Examiner 2. The root split is Tumor History; the negative Tumor History branch is subsequently partitioned by Boundary, with further refinement in the unclear Boundary branch using Facial Nerve Palsy and additional descriptors (e.g., Texture and related sonographic features). The positive Tumor History branch is refined primarily by Vascularization, with downstream splits including Nicotine consumption, Number of Tumors, Echogenicity, and Acoustic Features, reflecting examiner-specific decision-making tendencies; Figure S5: Decision-tree surrogate derived from Examiner 3’s assessments. This surrogate tree captures Examiner 3’s labeling behavior. The root split is Boundary, separating “sharp” and “unclear” branches. In the sharp-boundary branch, classification is refined primarily by Tumor History with additional downstream sonographic splits. In the unclear-boundary branch, the next discriminator is Contour, followed by further refinement using descriptors such as Echogenicity, Vascularization, and Acoustic Features, illustrating branch-dependent feature prioritization in this examiner’s decision pathway; Figure S6: Decision-tree surrogate derived from Examiner 4’s assessments. This examiner-specific surrogate reproduces Examiner 4’s binary calls. The root split is Boundary; the unclear Boundary branch is refined early by Facial Nerve Palsy and subsequent sonographic descriptors (e.g., Texture). The sharp Boundary branch is refined by Tumor History, with additional downstream splits including Nicotine consumption, Echogenicity, and Vascularization, providing an explicit hierarchical representation of this examiner’s decision logic; Figure S7: Decision-tree surrogate derived from Examiner 5’s assessments. This surrogate decision tree approximates Examiner 5’s labeling behavior. The root split is Boundary. The unclear Boundary branch is refined early by Facial Nerve Palsy and Number of Tumors, with downstream refinement using additional sonographic descriptors (e.g., Contour and Acoustic Features). The sharp Boundary branch is refined by Tumor History and further branch-specific splits (including clinical metadata such as Nicotine consumption and sonographic descriptors such as Echogenicity), reflecting examiner-specific pathways toward benign vs. malignant labels; Figure S8: Decision-tree surrogate derived from Examiner 6’s assessments. This surrogate tree was trained to reproduce Examiner 6’s binary labels. The root split is Boundary, with early divergence between the sharp Boundary branch (refined by Facial Nerve Palsy and Number of Tumors) and the unclear Boundary branch (refined by Acoustic Features). Additional downstream splits include Vascularization, Nicotine consumption, Echogenicity, Texture, and Tumor History, yielding a more extensively branched pathway consistent with this examiner’s labeling behavior. Table S1: Diagnostic performance of six examiners for binary classification of parotid tumors (malignant vs. benign) using histopathology as reference standard; Table S2: Misclassification patterns stratified by histopathological entity. For benign entities, values indicate false-positive classifications (cases labeled malignant) as *n*/*N* (%). For malignant cases (no malignancy subtyping available), values indicate false-negative classifications (cases labeled benign) as *n*/*N* (%). *N* reflects available cases per examiner (two examiners had *n *= 148 due to one missing case); Table S3: Global multi-rater agreement across all six examiners for each descriptor (Fleiss’ κ with 95% bootstrap confidence intervals; *n* = 147 complete cases); Table S4: Upper-tree structure (depth 0–1) of examiner-specific surrogate decision trees. For each examiner, the root split feature (depth 0) and the two primary branches (depth 1) are shown, including branch size and the subsequent split feature under each branch; Table S5: Pairwise similarity of examiner-specific surrogate decision trees (depth 0–2); Table S6: Structural complexity metrics of examiner-specific surrogate decision trees; Table S7: Association between examiner-specific surrogate decision tree complexity and examiner diagnostic performance relative to histopathology (malignant vs. benign); Table S8: Performance of examiner-specific surrogate decision trees with and without pruning.

## Abbreviations

The following abbreviations are used in this manuscript:

AI	artificial intelligence
AUC	area under the receiver operating characteristic curve
CDS	color-coded duplex sonography
CEUS	contrast-enhanced ultrasound
CI	confidence interval
DEGUM	Deutsche Gesellschaft für Ultraschall in der Medizin
ENT	ear, nose, and throat
NPV	negative predictive value
PPV	positive predictive value
ROC	receiver operating characteristic
US	ultrasound
XAI	explainable artificial intelligence
κ	Cohen’s kappa (kappa statistic)
