# Supplementary material for: Differential Diagnosis of Parotid Tumors on Ultrasound: Interobserver Variability and Examiner-Specific Decision Rules—A Machine Learning Approach
Source: Diagnostics (Basel). 2026 Mar 16;16(6):880. doi: 10.3390/diagnostics16060880 (PMC13025738; doi:10.3390/diagnostics16060880)
Supplement: Supplementary file 1 [file diagnostics-16-00880-s001.zip › Supplementary File S1.pdf]

Supplementary File S1. Descriptor-based sonographic profiles of parotid tumor entities

1. **Pleomorphic Adenoma (PA)**

• **Clinical Data:** It is the most common benign salivary gland tumor (60–80%) [1, 2]. It typically appears in patients in their **4th to 5th decade** of life [3] and shows a slight **female predominance** [4, 5] . Clinically, it presents as a slow-growing, painless, mobile, and usually unilateral mass [3]. There is a risk of malignant transformation (Carcinoma ex pleomorphic adenoma) in long-standing cases (1.5% after 5 years, 10% after 15 years) [5, 6]

• **B-Mode Features:** Typically **lobulated** or polycyclic in shape with sharp, well-defined margins [2, 7]. It is usually **hypoechoic** and homogeneous, though larger tumors (>3 cm) may become inhomogeneous due to necrosis or hemorrhage [1, 8].

• **Vascularization:** Often shows **low or only peripheral vascularity** [2, 9].

• **Other Parameters:** Frequently demonstrates **distal acoustic enhancement** [2, 9, 10]. Elastography usually shows **high stiffness** (high strain ratio) [9]. The Long-to-Short (L/S) diameter ratio is typically **< 1.5** [3].

2. **Warthin Tumor (Cystadenolymphoma)**

• **Clinical Data:** The second most common benign tumor [2, 11], most frequent in **elderly males** (6th to 7th decade) [2, 11, 12]. It is **strongly associated with smoking** (up to 94% of WT patients are smokers) [5, 6, 8]. In up to 20% of cases, it occurs **multifocally or bilaterally**. It is most commonly located in the **parotid tail** (inferior pole) [6, 10, 11, 13].

• **B-Mode Features:** Sharp margins, often **oval** in shape. The echotexture is typically inhomogeneous with **multiple small anechoic areas** ("sponge-like" pattern) [2, 11].

• **Vascularization:** Characteristically **strongly vascularized**, often with a central or hilar vessel distribution [2, 9, 14].

• **Other Parameters:** Generally shows **lower stiffness** in elastography compared to PA [9]. The L/S ratio is typically **> 1.5** ("slender" shape) [3].

3. **Oncocytoma**

• **Clinical Data:** A rare benign tumor of the parotid gland (~1%) [21] mainly affecting patients **over 60 years** old [1, 21]. **Local pain** can be a more frequent clinical symptom compared to other benign tumors [21].

• **B-Mode Features:** Sharp margins, often lobulated or irregular, and hypoechoic [9, 21]
• **Vascularization:** Typically **strongly vascularized** on Doppler (diffuse peripheral vascularization with some central vessels) [21]
• **Other Parameters:** Tends to show a **soft pattern** with low stiffness in elastography [9]

4. **Basal Cell Adenoma (BCA)**

• **Clinical Data:** Rare (1–3%), predominantly occurring in **females over 50–60 years** [4, 8, 22]. Usually located in the **superficial lobe** [4, 8] .

• **B-Mode Features:** Small (< 3 cm), round, and very **sharply defined**. Superficial tumors are solid; **cystic components** occur more often in deeper or larger tumors [8]

• **Other Parameters:** Shows **early, intense enhancement** in contrast-enhanced imaging [8]

5. Sialadenoses and Sialadenitis

• **Sialadenosis:** Chronic, painless, usually **bilateral** swelling [10]. Associated with **alcohol abuse, endocrine disorders** (Diabetes), malnutrition (**anorexia/bulimia**), or medications [10].

Ultrasound Features: Homogeneously **hyperechoic** (bright) gland enlargement [10].

• **Acute Sialadenitis:** Sudden pain, swelling, and **fever** [10].
Ultrasound Features: Enlarged, **diffusely inhomogeneous and hypoechoic**; strongly hypervascular [10].

• **Abscess:** Inhomogeneous, echo-free to hypoechoic zone with **blurred margins**; no internal vascularization within the fluid center [10].

Chronic Recurrent Parotitis: Shows a characteristic "honeycomb" or "leopard skin" pattern (Felix Johnson, Sonography of Salivary Gland Tumors and Disorders [10]

6. Malignancies (Primary)

General Clinical Information

• **Clinical Data:** Account for approximately 20% of parotid tumors [1], often in patients **over 60 years** old [15]. **Key clinical indicators** of malignancy include sudden growth, **facial nerve palsy** (highly suspicious), fixation to surrounding tissue, and pain (especially in Adenoid Cystic Carcinoma due to neurotropism) [1, 5, 6, 16].

• **Form & Margins:** Irregular shape and **unclear/infiltrative margins** are the most powerful predictors of malignancy [16].

• **Echogenicity & Texture:** Mostly **hypoechoic and inhomogeneous** [17].
Microcalcifications are more common in malignant nodules [16].
• **Vascularization:** Often **hypervascular** with a chaotic or scattered vessel pattern [9].
• **Other Parameters:** Presence of **pathological cervical lymph nodes** (round, loss of fatty hilum) [16]. High stiffness on elastography [9].

Specific Malignant Entities

6.1 Mucoepidermoid Carcinoma (MEC)

Clinical Data: MEC is the most common malignancy of the parotid gland. Its sonographic appearance varies significantly based on histological grade [17] .

• **Low-Grade MEC:** Often presents with **"benign-mimicking"** features, appearing well-defined with smooth borders and a homogeneous structure, making it difficult to distinguish from pleomorphic adenomas [9, 17].

• **High-Grade MEC:** Typically shows **ill-defined, infiltrative margins** and a highly heterogeneous internal architecture [9, 17].

• **Key Features:** In a study of 74 cases, 20.3% showed cystic areas, 12.2% contained calcifications, and 35.1% exhibited posterior acoustic enhancement—a feature often mistakenly associated only with benignity [17].

6.2 Acinic Cell Carcinoma (AciCC)

Clinical Data: AciCC is the third most common epithelial malignancy in adults and the second most common in children [18].

• **Sonographic Appearance:** Primarily **hypoechoic (73%)** and **heterogeneous (92%)**. While 60% of cases have an irregular shape, a significant majority **(79%) are well-defined**, which can lead to a false diagnosis of a benign tumor [18].

• **Vascularization:** Characteristically **poorly vascularized (83%)**, which helps differentiate it from highly vascularized Warthin tumors [18] .

6.3 Adenoid Cystic Carcinoma (ACC)

Clinical Data: ACC is known for its aggressive biological behavior and high propensity for **perineural invasion**.

• **Sonographic Trap:** Like low-grade MEC, ACC frequently mimics benign tumors by appearing as a well-defined, regular-shaped, homogeneous hypoechoic mass with posterior acoustic enhancement [19].

• **Elastography:** Despite its "innocent" B-mode appearance, ACC often exhibits **high stiffness** (higher strain ratio) compared to benign counterparts [19].

6.4 Salivary Duct Carcinoma (SDC)

Clinical Data: SDC is an uncommon but extremely high-grade and aggressive primary neoplasm [12].

• **US Appearance:** Presents as a voluminous, ill-defined hypoechoic mass with **multiple calcifications** (both clustered and dispersed) [12].
• **Vascularization:** Shows chaotic internal vascularization with **high resistance indices** (RI > 0.8 and PI > 2) [12].
• **Nodal Involvement:** High incidence of regional **malignant lymph node metastases (57–73%)** at the time of diagnosis [12].

6.5 Malignant Lymphoma

Clinical Data: Lymphoma manifestations are highly variable and can simulate both inflammatory and neoplastic processes [9, 10] .
• **US Pattern:** Often presents with a **pseudocystic or micronodular pattern** [9] .
• **Sjögren Syndrome Association:** In patients with Sjögren's, a poorly defined, hypoechoic mass with a **"cobblestone" or "honeycomb" pattern** should raise immediate suspicion for a MALT lymphoma [10].

6.6 Metastases

Clinical Data: The most common primary sites for parotid metastases are squamous cell carcinomas of the skin (scalp/ear) and melanomas.
• **US Appearance:** Usually manifests as **enlarged intraparotid lymph nodes**.
• **Malignancy Markers:** Round shape, loss of the typical echogenic fatty hilum, and disorganized/chaotic vascularity [20]

7. Systemic Diseases (Sjögren Syndrome and HIV)

• **Sjögren Syndrome:** Autoimmune, mostly in **post-menopausal females** [1]. Clinical **sicca symptoms** (dry eyes/mouth)**.** High risk for **MALT lymphoma** [10].
US: **Bilateral**, diffuse **"honeycomb"** or **"leopard skin"** pattern due to multiple small hypoechoic areas [9, 10].

• **HIV-Associated Lesions:** Multiple, **bilateral cystic and solid** changes (benign lymphoepithelial lesions) [1, 10]. Often accompanied by persistent cervical lymphadenopathy [1].

8. Others (Lymph Nodes, Cysts, Lipomas)

• **Lymph Nodes:** Intraparotid nodes are physiologically normal (up to 10) [6].
Benign: oval, hypoechoic with **echogenic central hilus** [1].
Malignant nodes (metastases): round, loss of hilus, chaotic vascularity [1, 20].

• **Cysts:** Anechoic, sharply defined, with **distal acoustic enhancement** [1, 10] . In adults, a solitary cyst must be distinguished from a **cystic lymph node metastasis** [6] .

• **Lipomas:** Well-defined, isoechoic to fat or hypoechoic with **characteristic feathery, echogenic striae** [1] soft on elastography [9].

References:

1. Lee YYP, Wong KT, King AD, Ahuja AT (2008) Imaging of salivary gland tumours. Eur J Radiol 66:419–436. https://doi.org/10.1016/j.ejrad.2008.01.027
2. Rong X, Zhu Q, Ji H, et al (2013) Differentiation of pleomorphic adenoma and Warthin's tumor of the parotid gland: ultrasonographic features. Acta Radiol 55:1203–1209. https://doi.org/10.1177/0284185113515865

3. Miao L-Y, Xue H, Ge H-Y, et al (2015) Differentiation of pleomorphic adenoma and Warthin's tumour of the salivary gland: is long-to-short diameter ratio a useful parameter? Clin Radiol 70:1212–1219. https://doi.org/10.1016/j.crad.2015.06.085
4. Chiu N-C, Wu H-M, Chou Y-H, et al (2007) Basal Cell Adenoma Versus Pleomorphic Adenoma of the Parotid Gland: CT Findings. Am J Roentgenol 189:W254–W261. https://doi.org/10.2214/ajr.07.2292
5. Kuang J, Rao Q, Cheng Z (2025) Diagnostic accuracy of ultrasound and MRI in parotid gland tumors: A retrospective study. Exp Ther Med 30:170. https://doi.org/10.3892/etm.2025.12920
6. Rigsby RK, Thoroughman OA, Tomblinson CM (2025) Review of the Parotid Space: Imaging Features and Diagnostic Pearls. Semin Ultrasound, CT MRI 46:344–356. https://doi.org/10.1053/j.sult.2025.09.008
7. Zajkowski P, Jakubowski W, Białek EJ, et al (2000) Pleomorphic adenoma and adenolymphoma in ultrasonography. Eur J Ultrasound 12:23–29. https://doi.org/10.1016/s0929-8266(00)00096-3
8. Shi L, Wang Y-XJ, Yu C, et al (2012) CT and Ultrasound Features of Basal Cell Adenoma of the Parotid Gland: A Report of 22 Cases with Pathologic Correlation. Am J Neuroradiol 33:434–438. https://doi.org/10.3174/ajnr.a2807
9. Martino M, Fodor D, Fresilli D, et al (2020) Narrative review of multiparametric ultrasound in parotid gland evaluation. Gland Surg 9:2295311–2292311. https://doi.org/10.21037/gs-20-530
10. Johnson F, Bozzato A, Mansour N, et al (2024) Sonography of Salivary Gland Tumors and Disorders. Ultraschall Med - Eur J Ultrasound 46:318–344. https://doi.org/10.1055/a-2481-7248
11. Kim J, Kim E, Park CS, et al (2004) Characteristic sonographic findings of Warthin's tumor in the parotid gland. J Clin Ultrasound 32:78–81. https://doi.org/10.1002/jcu.10230
12. Fenesan DI, Lenghel M, Baciut G, et al (2015) Ultrasound and CT imaging features in a patient with salivary duct carcinoma of the parotid gland: a case report with literature review. Méd Ultrason 17:119–122. https://doi.org/10.11152/mu.2013.2066.171.dif
13. Berner F, Koch M, Müller SK, et al (2021) Investigation of Sonographic Criteria for Reliable Identification of T1–T2 Low-Grade Malignant Tumors of the Parotid Gland. Ultrasound Med Biol 47:471–477. https://doi.org/10.1016/j.ultrasmedbio.2020.11.010
14. Knopf A, Mansour N, Chaker A, et al (2012) Multimodal ultrasonographic characterisation of parotid gland lesions—A pilot study. Eur J Radiol 81:3300–3305. https://doi.org/10.1016/j.ejrad.2012.01.004
15. Reginelli A, Clemente A, Renzulli M, et al (2019) Delayed enhancement in differential diagnosis of salivary gland neoplasm. Gland Surg 0:S130–S135. https://doi.org/10.21037/gs.2019.03.03
16. Gao T, Lin Y, Li W, et al (2024) Prediction of malignant risk stratification model for parotid gland nodules based on clinical and conventional ultrasound features: construction and validation. Gland Surg 13:1229242–1221242. https://doi.org/10.21037/gs-24-119
17. Gong X, Xiong P, Liu S, et al (2012) Ultrasonographic appearances of mucoepidermoid carcinoma of the salivary glands. Oral Surg, Oral Med, Oral Pathol Oral Radiol 114:382–387. https://doi.org/10.1016/j.oooo.2012.04.014
18. Li J, Gong X, Xiong P, et al (2014) Ultrasound and computed tomography features of primary acinic cell carcinoma in the parotid gland: A retrospective study. Eur J Radiol 83:1152–1156. https://doi.org/10.1016/j.ejrad.2014.03.017
19. Zeng X, Duan H, Ma X (2025) A case report of misdiagnosed adenoid cystic carcinoma of the parotid gland on ultrasound: imaging-pathological correlation and diagnostic implications. Gland Surg 14:2528534–2522534. https://doi.org/10.21037/gs-2025-354

20. Sriskandan N, Hannah A, Howlett DC (2010) A study to evaluate the accuracy of ultrasound in the diagnosis of parotid lumps and to review the sonographic features of parotid lesions — results in 220 patients. Clin Radiol 65:366–372. https://doi.org/10.1016/j.crad.2010.01.009
21. Lv K, Cao X, Geng D, Zhang J (2021) Imaging features of parotid gland oncocytoma: a case series study. Gland Surg 10:87076–87876. https://doi.org/10.21037/gs-20-790
22. Knipe H, Bickle I, Basal cell adenoma. Reference article, Radiopaedia.org (Accessed on 10 Mar 2026) https://doi.org/10.53347/rID-70088
