# Supplementary material for: Differential Diagnosis of Parotid Tumors on Ultrasound: Interobserver Variability and Examiner-Specific Decision Rules—A Machine Learning Approach
Source: Diagnostics (Basel). 2026 Mar 16;16(6):880. doi: 10.3390/diagnostics16060880 (PMC13025738; doi:10.3390/diagnostics16060880)
Supplement: Supplementary file 1 [file diagnostics-16-00880-s001.zip › Supplementary Table S1.pdf]

Supplementary Table S1. Diagnostic performance of six examiners for binary classification of parotid tumors (malignant vs. benign) using histopathology as reference standard.

Examiner	n	TP	TN	FP	FN	Sensitivity, % (95% CI)	Specificity, % (95% CI)	PPV, %	NPV, %	Accuracy, % (95% CI)	AUC (95% CI)
**Examiner 1**	149	34	87	15	13	72.3 (58.2–83.1)	85.3 (77.1–90.9)	69.4	87.0	81.2 (74.2–86.7)	0.79 (0.71–0.86)
**Examiner 2**	149	32	89	13	15	68.1 (53.8–79.6)	87.3 (79.4–92.4)	71.1	85.6	81.2 (74.2–86.7)	0.78 (0.70–0.85)
**Examiner 3**	148	40	94	7	7	85.1 (72.3–92.6)	93.1 (86.4–96.6)	85.1	93.1	90.5 (84.7–94.3)	0.89 (0.83–0.95)
**Examiner 4**	149	35	81	21	12	74.5 (60.5–84.7)	79.4 (70.6–86.1)	62.5	87.1	77.9 (70.5–83.8)	0.77 (0.70–0.84)
**Examiner 5**	149	34	66	36	13	72.3 (58.2–83.1)	64.7 (55.1–73.3)	48.6	83.5	67.1 (59.2–74.1)	0.69 (0.61–0.76)
**Examiner 6**	148	34	60	41	13	72.3 (58.2–83.1)	59.4 (49.7–68.5)	45.3	82.2	63.5 (55.5–70.8)	0.66 (0.58–0.74)

Abbreviations: AUC: area under the receiver operating characteristic curve; CI: confidence interval; FN: false negatives; FP: false positives; NPV: negative predictive value; PPV: positive predictive value; TN: true negatives; TP: true positives.
