# Supplementary material for: Differential Diagnosis of Parotid Tumors on Ultrasound: Interobserver Variability and Examiner-Specific Decision Rules—A Machine Learning Approach
Source: Diagnostics (Basel). 2026 Mar 16;16(6):880. doi: 10.3390/diagnostics16060880 (PMC13025738; doi:10.3390/diagnostics16060880)
Supplement: Supplementary file 1 [file diagnostics-16-00880-s001.zip › Supplementary Table S2.pdf]

Supplementary Table S2. Misclassification patterns stratified by histopathological entity.

For benign entities, values indicate false-positive classifications (cases labeled malignant) as n/N (%). For malignant cases (no malignancy subtyping available), values indicate false-negative classifications (cases labeled benign) as n/N (%). N reflects available cases per examiner (two examiners had n=148 due to one missing case).

A) Benign entities: false positives (labeled malignant)

Histopathological entity (benign)	Cohort n	Examiner 1	Examiner 2	Examiner 3	Examiner 4	Examiner 5	Examiner 6
**Warthin Tumor**	41	3/41 (7.3%)	3/41 (7.3%)	1/41 (2.4%)	7/41 (17.1%)	14/41 (34.1%)	23/41 (56.1%)
**Pleomorphic Adenoma**	34	7/34 (20.6%)	5/34 (14.7%)	3/33 (9.1%)	7/34 (20.6%)	11/34 (32.4%)	11/34 (32.4%)
**other**	8	1/8 (12.5%)	1/8 (12.5%)	0/8 (0.0%)	1/8 (12.5%)	2/8 (25.0%)	1/8 (12.5%)
**Sialadenitis**	8	3/8 (37.5%)	3/8 (37.5%)	3/8 (37.5%)	3/8 (37.5%)	4/8 (50.0%)	2/7 (28.6%)
**Lymph node**	7	1/7 (14.3%)	1/7 (14.3%)	0/7 (0.0%)	3/7 (42.9%)	4/7 (57.1%)	3/7 (42.9%)
**Cyst**	3	0/3 (0.0%)	0/3 (0.0%)	0/3 (0.0%)	0/3 (0.0%)	1/3 (33.3%)	1/3 (33.3%)
**Angioma**	1	0/1 (0.0%)	0/1 (0.0%)	0/1 (0.0%)	0/1 (0.0%)	0/1 (0.0%)	0/1 (0.0%)

B) Malignant cases: false negatives (labeled benign)

Histopathological entity (malignant)	Cohort n	Examiner 1	Examiner 2	Examiner 3	Examiner 4	Examiner 5	Examiner 6
**malignant**	47	13/47 (27.7%)	15/47 (31.9%)	7/47 (14.9%)	12/47 (25.5%)	13/47 (27.7%)	13/47 (27.7%)
