# Supplementary material for: Differential Diagnosis of Parotid Tumors on Ultrasound: Interobserver Variability and Examiner-Specific Decision Rules—A Machine Learning Approach
Source: Diagnostics (Basel). 2026 Mar 16;16(6):880. doi: 10.3390/diagnostics16060880 (PMC13025738; doi:10.3390/diagnostics16060880)
Supplement: Supplementary file 1 [file diagnostics-16-00880-s001.zip › Supplementary Table S3.pdf]

Supplementary Table S3. Global multi-rater agreement across all six examiners for each descriptor (Fleiss' κ with 95% bootstrap confidence intervals; n = 147 complete cases).

**Descriptor**	**Fleiss' κ**	**95% CI**
Malignancy Assessment (benign vs. malignant)	0.48	0.40–0.56
Boundary	0.62	0.55–0.69
Contour	0.44	0.38–0.50
Echogenicity	0.27	0.20–0.33
Texture	0.49	0.41–0.57
Size	0.77	0.71–0.82
Vascularization	0.38	0.30–0.45
Acoustic Features	0.45	0.37–0.52
Number of Tumors	0.56	0.47–0.64
