# Supplementary material for: Differential Diagnosis of Parotid Tumors on Ultrasound: Interobserver Variability and Examiner-Specific Decision Rules—A Machine Learning Approach
Source: Diagnostics (Basel). 2026 Mar 16;16(6):880. doi: 10.3390/diagnostics16060880 (PMC13025738; doi:10.3390/diagnostics16060880)
Supplement: Supplementary file 1 [file diagnostics-16-00880-s001.zip › Supplementary Table S4.pdf]

Supplementary Table S4. Upper-tree structure (depth 0–1) of examiner-specific surrogate decision trees. For each examiner, the root split feature (depth 0) and the two primary branches (depth 1) are shown, including branch size and the subsequent split feature under each branch.

Examiner	Root split feature (depth 0)	Branch condition (depth 1)	Branch size, n (%)	Next split feature (depth 1)
**Examiner 1**	Tumor History	Tumor History = no	114 (76.5%)	Boundary
**Examiner 1**	Tumor History	Tumor History = yes	35 (23.5%)	Vascularization
**Examiner 2**	Tumor History	Tumor History = no	114 (76.5%)	Boundary
**Examiner 2**	Tumor History	Tumor History = yes	35 (23.5%)	Vascularization
**Examiner 3**	Boundary	Boundary = sharp	100 (67.6%)	Tumor History
**Examiner 3**	Boundary	Boundary = unclear	48 (32.4%)	Contour
**Examiner 4**	Boundary	Boundary = sharp	94 (63.1%)	Tumor History
**Examiner 4**	Boundary	Boundary = unclear	55 (36.9%)	Facial Nerve Palsy
**Examiner 5**	Boundary	Boundary = sharp	89 (59.7%)	Tumor History
**Examiner 5**	Boundary	Boundary = unclear	60 (40.3%)	Facial Nerve Palsy
**Examiner 6**	Boundary	Boundary = sharp	101 (68.2%)	Facial Nerve Palsy
**Examiner 6**	Boundary	Boundary = unclear	47 (31.8%)	Acoustic Features
