# Supplementary material for: Differential Diagnosis of Parotid Tumors on Ultrasound: Interobserver Variability and Examiner-Specific Decision Rules—A Machine Learning Approach
Source: Diagnostics (Basel). 2026 Mar 16;16(6):880. doi: 10.3390/diagnostics16060880 (PMC13025738; doi:10.3390/diagnostics16060880)
Supplement: Supplementary file 1 [file diagnostics-16-00880-s001.zip › Supplementary Table S5.pdf]

Supplementary Table S5. Pairwise similarity of examiner-specific surrogate decision trees (depth 0–2).
(**A**) Path-context overlap (Jaccard index).
Jaccard = |intersection of path contexts| / |union of path contexts|

	**Examiner 1**	**Examiner 2**	**Examiner 3**	**Examiner 4**	**Examiner 5**	**Examiner 6**
**Examiner 1**	1.00	0.83	0.08	0.09	0.09	0.09
**Examiner 2**	0.83	1.00	0.08	0.10	0.10	0.10
**Examiner 3**	0.08	0.08	1.00	0.56	0.56	0.27
**Examiner 4**	0.09	0.10	0.56	1.00	1.00	0.33
**Examiner 5**	0.09	0.10	0.56	1.00	1.00	0.33
**Examiner 6**	0.09	0.10	0.27	0.33	0.33	1.00

(**B**) RecordCount-weighted split agreement on shared path contexts.

	**Examiner 1**	**Examiner 2**	**Examiner 3**	**Examiner 4**	**Examiner 5**	**Examiner 6**
**Examiner 1**	1.00	0.83	0.00	0.00	0.00	0.00
**Examiner 2**	0.83	1.00	0.00	0.00	0.00	0.00
**Examiner 3**	0.00	0.00	1.00	0.62	0.62	0.50
**Examiner 4**	0.00	0.00	0.62	1.00	0.68	0.50
**Examiner 5**	0.00	0.00	0.62	0.68	1.00	0.50
**Examiner 6**	0.00	0.00	0.50	0.50	0.50	1.00
