# Supplementary material for: Differential Diagnosis of Parotid Tumors on Ultrasound: Interobserver Variability and Examiner-Specific Decision Rules—A Machine Learning Approach
Source: Diagnostics (Basel). 2026 Mar 16;16(6):880. doi: 10.3390/diagnostics16060880 (PMC13025738; doi:10.3390/diagnostics16060880)
Supplement: Supplementary file 1 [file diagnostics-16-00880-s001.zip › Supplementary Table S6.pdf]

Supplementary Table S6. Structural complexity metrics of examiner-specific surrogate decision trees.

Examiner	Total number of nodes	Number of internal (split) nodes	Number of terminal nodes (leaves)	Maximum depth (edges)	Mean leaf depth	RecordCount-weighted mean leaf depth	Number of unique split features
**Examiner 1**	66	28	38	10	5.79	4.32	10
**Examiner 2**	67	28	39	9	6.05	3.78	10
**Examiner 3**	75	33	42	8	5.48	4.93	10
**Examiner 4**	64	27	37	10	5.68	4.31	10
**Examiner 5**	64	28	36	10	5.97	5.15	10
**Examiner 6**	105	45	60	10	6.48	5.78	10
