# Supplementary material for: Differential Diagnosis of Parotid Tumors on Ultrasound: Interobserver Variability and Examiner-Specific Decision Rules—A Machine Learning Approach
Source: Diagnostics (Basel). 2026 Mar 16;16(6):880. doi: 10.3390/diagnostics16060880 (PMC13025738; doi:10.3390/diagnostics16060880)
Supplement: Supplementary file 1 [file diagnostics-16-00880-s001.zip › Supplementary Table S7.pdf]

Supplementary Table S7. Association between examiner-specific surrogate decision tree complexity and examiner diagnostic performance relative to histopathology (malignant vs. benign).

Tree complexity measure	Definition	Balanced accuracy, Spearman's ϱ	p-value	Sensitivity, Spearman's ϱ	p-value	Specificity, Spearman's ϱ	p-value
**Total number of tree nodes**	Total count of nodes (internal + terminal nodes)	0.058	0.933	-0.031	0.983	0.116	0.844
**Number of terminal nodes (leaves)**	Total count of terminal nodes	0.086	0.919	0.030	1.000	0.143	0.803
**Maximum tree depth**	Maximum root-to-leaf path length (edges)	-0.676	0.200	-0.180	0.867	-0.845	0.067
**Record Count-weighted mean leaf depth**	Mean leaf depth weighted by PMML node Record Count (reflecting case-supported path depth)	-0.486	0.356	0.213	0.733	-0.600	0.242

Abbreviations: PMML, Predictive Model Markup Language.
Notes: Correlations were assessed using two-sided Spearman rank correlation (n = 6 examiners). p-values are exact permutation-based p-values. Positive class was defined as **malignancy**. Tree complexity metrics were extracted from examiner-specific surrogate decision trees exported as PMML from KNIME.
