# Supplementary material for: Differential Diagnosis of Parotid Tumors on Ultrasound: Interobserver Variability and Examiner-Specific Decision Rules—A Machine Learning Approach
Source: Diagnostics (Basel). 2026 Mar 16;16(6):880. doi: 10.3390/diagnostics16060880 (PMC13025738; doi:10.3390/diagnostics16060880)
Supplement: Supplementary file 1 [file diagnostics-16-00880-s001.zip › Supplementary Table S8.pdf]

Supplementary Table S8. Performance of examiner-specific surrogate decision trees with and without pruning.

Table S8A. Performance vs. histopathology; McNemar-Test compares pruned vs unpruned (Holm-corrected across examiners

Examiner	Endpoint	Model	n total	n valid	Coverage	Accuracy	Sensitivity	Specificity	PPV	NPV	Balanced Accuracy	Cohen's kappa (95% CI)	n paired	McNemar-p Value
**1**	Histology	unpruned	149	145	97.3	80.0	76.1	81.8	66.0	88.0	79.0	0.56 (0.41-0.69)		
**1**	Histology	pruned	149	149	100.0	73.8	57.4	81.4	58.7	80.6	69.4	0.39 (0.22-0.55)	145	0.6802
**2**	Histology	unpruned	149	147	98.7	78.2	60.9	86.1	66.7	82.9	73.5	0.48 (0.32-0.63)		
**2**	Histology	pruned	149	149	100.0	76.5	53.2	87.3	65.8	80.2	70.2	0.43 (0.26-0.58)	147	1.0000
**3**	Histology	unpruned	148	141	95.3	77.3	62.8	83.7	62.8	83.7	73.2	0.46 (0.29-0.62)		
**3**	Histology	pruned	148	148	100.0	76.4	63.8	82.2	62.5	83.0	73.0	0.46 (0.30-0.61)	141	1.0000
**4**	Histology	unpruned	149	146	98.0	76.0	66.7	80.2	60.0	84.4	73.4	0.45 (0.30-0.60)		
**4**	Histology	pruned	149	149	100.0	75.2	80.9	72.5	57.6	89.2	76.7	0.48 (0.34-0.62)	146	1.0000
**5**	Histology	unpruned	149	143	96.0	70.6	77.3	67.7	51.5	87.0	72.5	0.39 (0.25-0.55)		
**5**	Histology	pruned	149	149	100.0	65.1	63.8	65.7	46.2	79.8	64.8	0.27 (0.12-0.42)	143	0.6802
**6**	Histology	unpruned	148	145	98.0	57.2	60.9	55.6	38.9	75.3	58.2	0.14 (-0.01-0.28)		
**6**	Histology	pruned	148	148	100.0	73.6	74.5	73.3	56.5	86.0	73.9	0.44 (0.28-0.59)	145	0.0007

Table S8B. Fidelity vs examiner labels; McNemar-Test compares pruned vs unpruned (Holm-corrected across examiners).

Examiner	Endpoint	Model	n total	n valid	Coverage	Accuracy	Sensitivity	Specificity	PPV	NPV	Balanced Accuracy	Cohen's kappa (95% CI)	n paired	McNemar-p Value
**1**	Fidelity	unpruned	149	145	97.3	80.7	76.6	82.7	67.9	88.0	79.6	0.57 (0.43-0.71)		
**1**	Fidelity	pruned	149	149	100.0	76.5	61.2	84.0	65.2	81.6	72.6	0.46 (0.31-0.61)	145	1.0000
**2**	Fidelity	unpruned	149	147	98.7	78.2	61.4	85.4	64.3	83.8	73.4	0.47 (0.31-0.62)		
**2**	Fidelity	pruned	149	149	100.0	80.5	60.0	89.4	71.1	83.8	74.7	0.52 (0.35-0.66)	147	1.0000
**3**	Fidelity	unpruned	148	141	95.3	81.6	69.8	86.7	69.8	86.7	78.3	0.57 (0.41-0.71)		
**3**	Fidelity	pruned	148	148	100.0	84.5	76.6	88.1	75.0	89.0	82.4	0.64 (0.50-0.77)	141	1.0000
**4**	Fidelity	unpruned	149	146	98.0	83.6	74.1	89.1	80.0	85.4	81.6	0.64 (0.50-0.77)		
**4**	Fidelity	pruned	149	149	100.0	85.2	89.3	82.8	75.8	92.8	86.0	0.70 (0.57-0.81)	146	1.0000
**5**	Fidelity	unpruned	149	143	96.0	82.5	81.5	83.3	80.3	84.4	82.4	0.65 (0.52-0.77)		
**5**	Fidelity	pruned	149	149	100.0	85.9	81.4	89.9	87.7	84.5	85.7	0.72 (0.60-0.82)	143	1.0000
**6**	Fidelity	unpruned	148	145	98.0	70.3	69.9	70.8	70.8	69.9	70.3	0.41 (0.25-0.55)		
**6**	Fidelity	pruned	148	148	100.0	73.6	65.3	82.2	79.0	69.8	73.8	0.47 (0.32-0.61)	145	1.0000

Notes: Cohen's kappa is unweighted (binary). 95% confidence intervals were obtained by nonparametric bootstrap resampling of cases (2,000 iterations). Missing predictions (unpruned) were excluded; coverage is reported separately. McNemar tests compare pruned vs. unpruned on paired cases and are exact, Holm-corrected across examiners within each endpoint.
