# Supplementary figures and images for: Differential Diagnosis of Parotid Tumors on Ultrasound: Interobserver Variability and Examiner-Specific Decision Rules—A Machine Learning Approach
Source: Diagnostics (Basel). 2026 Mar 16;16(6):880. doi: 10.3390/diagnostics16060880 (PMC13025738; doi:10.3390/diagnostics16060880)

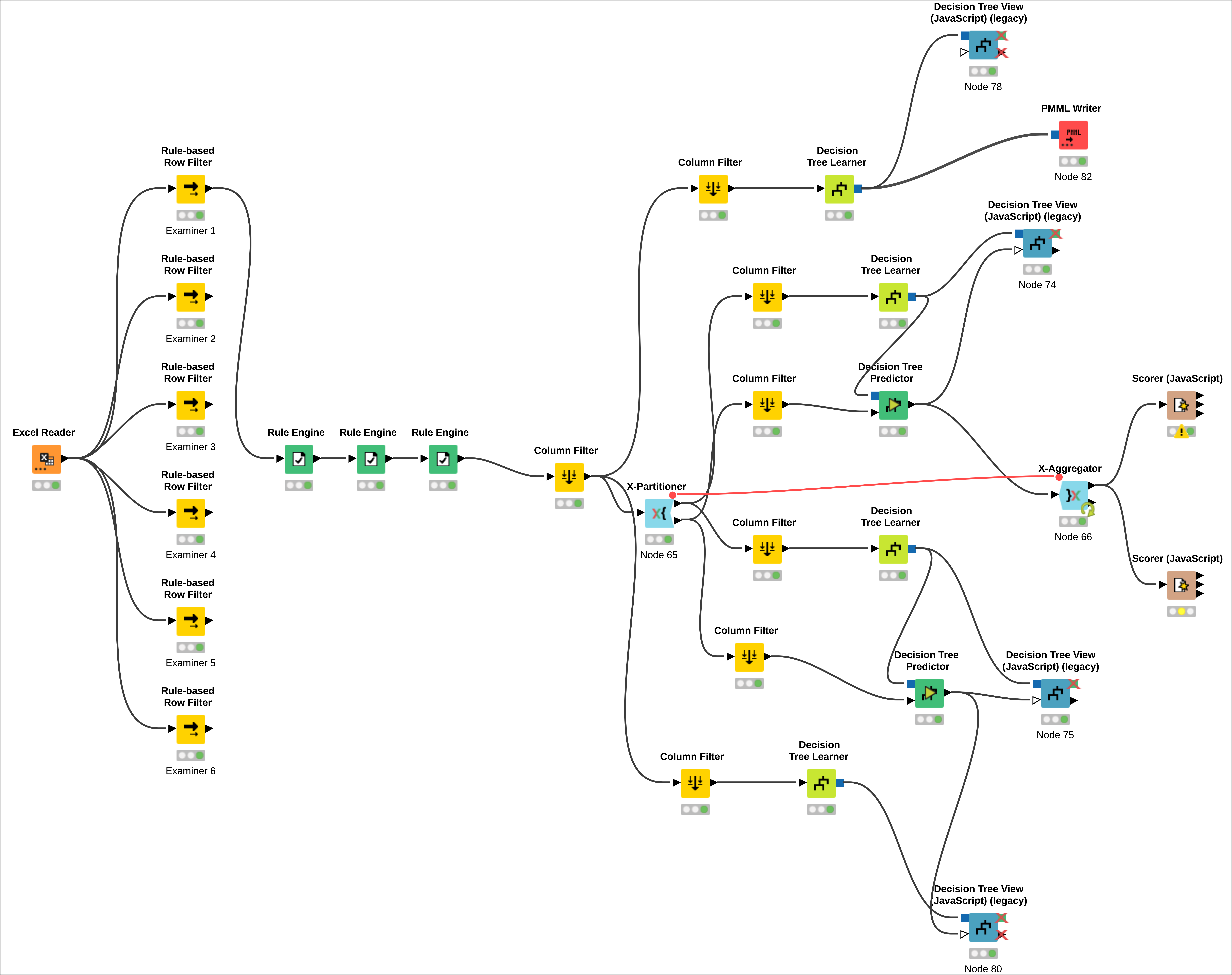

Supplement: Supplementary file 1 [file diagnostics-16-00880-s001.zip › Supplementary Figure_S2.png]
